# Supplementary figures and images for: Lack of an association between clinical INSTI-related body weight gain and direct interference with MC4 receptor (MC4R), a key central regulator of body weight
Source: PLoS One. 2020 Feb 28;15(2):e0229617. doi: 10.1371/journal.pone.0229617 (PMC7048285; doi:10.1371/journal.pone.0229617)

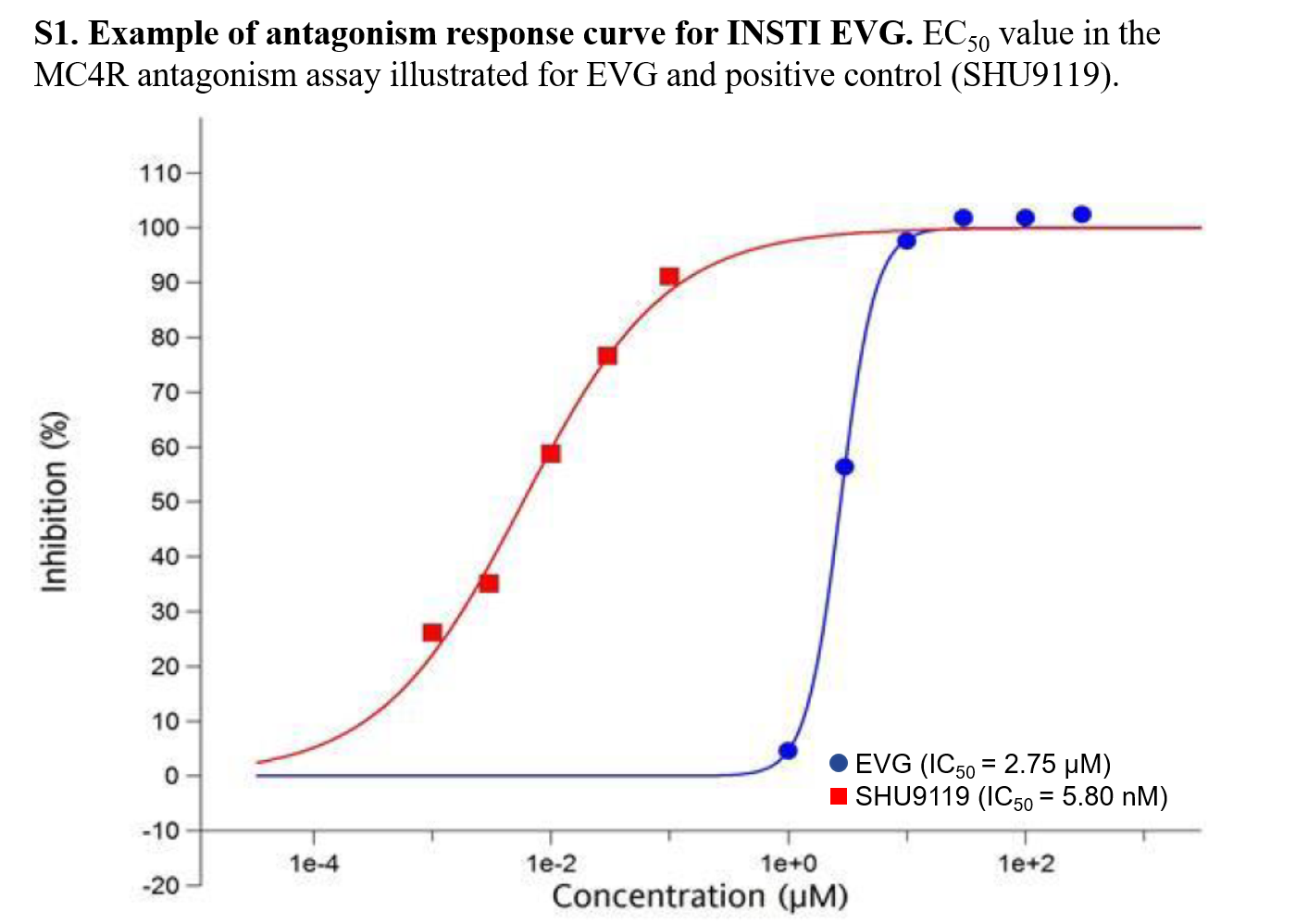

Supplement: S1 Fig — EC50 value in the MC4R antagonism assay illustrated for EVG and positive control (SHU9119). (TIF) [file pone.0229617.s001.tif]
