# Supplementary material for: Lack of an association between clinical INSTI-related body weight gain and direct interference with MC4 receptor (MC4R), a key central regulator of body weight
Source: PLoS One. 2020 Feb 28;15(2):e0229617. doi: 10.1371/journal.pone.0229617 (PMC7048285; doi:10.1371/journal.pone.0229617)
Supplement: S1 Table — (DOCX) [file pone.0229617.s002.docx]

**Supporting Information**

**S1. Biochemical binding assay**

| **Compound** | **% binding at 100 µM** | **IC_50_ (µM)** |
| --- | --- | --- |
| BIC | 89 | 4.9 |
| DTG | 91 | 1.7 |
| CAB | 86 | 0.46 |
| EVG | 56 | 13 |
| RAL | 55 | 78 |

Note: Reference compound NDP-α-MSH IC_50_=0.00029 µM
