# Supplementary material for: Lack of an association between clinical INSTI-related body weight gain and direct interference with MC4 receptor (MC4R), a key central regulator of body weight
Source: PLoS One. 2020 Feb 28;15(2):e0229617. doi: 10.1371/journal.pone.0229617 (PMC7048285; doi:10.1371/journal.pone.0229617)
Supplement: S2 Table — (DOCX) [file pone.0229617.s003.docx]

**Supporting Information**

**S2. Human C_max_ values for approved INSTIs at recommended clinical dosages**

| **Compound** | **Clinical dosage** | **Human efficacious C_max_**  **(µM)** | **% unbound** | **Human efficacious unbound C_max_**  **(µM)** |
| --- | --- | --- | --- | --- |
| BIC | 50 mg | 13.7 | 0.25% | 0.034 |
| DTG | 50 mg | 8.8 | 0.7% | 0.062 |
| CAB | 30 mg | 15.7 | 0.16% | 0.025 |
| EVG | 150 mg  (+150 mg cobicistat) | 4.7 | 0.48% | 0.0225 |
| RAL | 1200 mg | 15.7 | 12.2% | 1.9 |
